# Evaluation of applied public health emergency system at Prince Mohammed International Airport in Almedinah during Hajj season 2014: a qualitative case study

**DOI:** 10.1186/s13104-015-1415-2

**Published:** 2015-09-12

**Authors:** Ibrahim M. Gosadi, Abdulaziz BinSaeed, Ali M. Al-Hazmi, Amin A. Fadl, Khalid H. Alharbi, Mazin M. Swarelzahab

**Affiliations:** Prince Sattam Chair for Epidemiology and Public Health Research, Community Medicine Unit, College of Medicine, King Saud University, BO 2454, Riyadh, 11541 Saudi Arabia; Ministry of Health, Riyadh, Saudi Arabia; Department of Family and Community Medicine, College of Medicine, King Saud University, Riyadh, Saudi Arabia

**Keywords:** Emergency, Public, Health, Hajj, Preventive, Airport

## Abstract

**Background:**

During the Hajj season 2014, several public health measures were applied by the Ministry of Health at Prince Mohammed International Airport in Almedinah. However, several operational defects affected the provision of preventive health services for passengers and airport workers. This study aims to evaluate the applied public health emergency system at the airport, detect any potential gaps and to provide appropriate operational solutions.

**Methods:**

This is a qualitative case study conducted at Prince Mohammed International Airport in Almedinah during the 2014 Hajj season, September 2014. Data were collected via semi-structured interviews, focus groups and policy document reviews. Interviews were conducted with the 14 individuals of the airport’s decision makers and relevant health practitioners. Data were recorded via taking notes during interviews and data coding was performed to produce the main themes and subthemes of the study.

**Results:**

The main findings of the study revealed three main defects affecting the applied public health emergency system at the airport. The main themes were mainly related to shortage in logistics related to public health emergency systems, shortage in proper documentation of policies and lack of documented protocols of communications among airport stakeholders.

**Conclusions:**

The study highlighted the main factors hindering the application of public health emergency measures at the airport. A Public Health Emergency Contingency Plan was proposed as a method to regulate the process of providing logistics for public health preventive services, the method of producing documented policies and methods of producing Memoranda of Understandings as communication regulators.

## Background

The development of transportation systems has increased the global movement of individuals across continents. With the increased movement comes the higher risk of emerging communicable diseases spreading on an international scale. The normal movement of passengers is sufficient to increase the risk of communicable diseases. Gathering of large number of people in a densely occupied space within a limited time period and then quickly dispersing throughout the world is likely to increase the risk of developing epidemics.

Most of the international movements take place through airports. Points of entry, including airports, should maintain a proper public health emergency system to ensure proper preparedness for any potential public health events, to enhance any interventional efforts and to ensure a coordinated application of public health measures with minimum conflicts between airport workers [1]. If no such measures are taken, passengers and airport workers will not be properly protected against emerging communicable diseases and will be susceptible for acquiring such diseases. Such susceptibility might even have a stronger impact on increasing the spread of communicable diseases in populations.

During Hajj season 2014, several public health measures were applied by the Ministry of Health. These measures were performed by the airport health care workers including screening measures for communicable diseases, provision of vaccination and prophylaxis for pilgrims of certain countries and provision of medical therapeutic services [2]. However, several conflicting incidents occurred during provision of preventive and curative health services for arriving pilgrims to Prince Mohammed International Airport in Almedinah. Conflicts included hindering flow of passengers and provision of preventive and curative health services. Ramifications of the conflicts included failing to give appropriate vaccinations and prophylaxis to arriving pilgrims of certain countries and failure to provide medical advice and procedures when needed.

This qualitative investigation aims to perform a situational analysis of the public health emergency system applied at the airport, to thoroughly investigate potential reasons for conflicts among airport workers, and to investigate possible methods of enhancing the applied system. Additional objectives of this study include highlighting areas of weakness and shortage of applied protocols and guidelines related to public health emergencies at the airport. The study recommendations will enable the airport administration to identify gaps in knowledge, guidelines, communications and skills relating to public health emergencies and act upon solving these issues.

## Methods

This study is a qualitative case study undertaken at Prince Mohammed Bin Abdul Aziz International Airport in Almedinah during the Hajj season of 2014. Data was collected through semi-structured interviews, focus group, and a review of health policies implemented at the airport. All of the participants were aged above 18 and were permanent workers at the airport at the time of data collection. Fourteen individuals were recruited in this investigation.

Purposive selection was conducted to recruit key informants. The key informants were stakeholders at the airport, including the general manager of the airport, relevant managers of governmental sectors and relevant managers of private companies operating the airport. Only informative stakeholders were included in this study as they were more likely to provide key information about the public health operational situations and to be more involved with policy making. Individuals who were not relevant stakeholders at the airport were excluded. Personal site visits and contact with subjects via telephones were used to approach the participants. All of the subjects who were approached agreed to participate in the study.

Two authors conducted the interviews: IG (male), a consultant epidemiologist with previous research experience and training and KH (male), a consultant family physician with experience in clinical health systems. Policy reviewing was conducted by IG and MS (male consultant epidemiologist with a background in infection control systems). All the investigators were not workers at the airport, and relationships between interviewers and participants were not established before the interviews.

The focus group was facilitated by IG and KH with no other independent observer. No audio recording was performed and only handwritten notes were collected. The focus group included 7 health care workers who were permanent workers at the airport. It included local medical supervisor, epidemiologists, nursing supervisor, and medical logistics coordinators. All health care workers were employed by the Saudi Ministry of Health.

Participants were introduced to the interviewers and provided with a basic background of the study and the training of the researchers prior to the interviews. Interviews and the focus group were conducted at the airport. Each participant was only interviewed once and responses were recorded via handwritten notes. A summary of the notes was reviewed with the participants at the end of each interviewing session. No transcripts were presented to the participants for additional comments. All interviews were conducted in Arabic and were translated to English before data analysis. The translation was conducted by IG who is a native Arabic speaker and fluent in English.

Interview questions were adopted from the International Health Regulation Assessment Tool for Points of Entry [3]. Questions were mainly related to communication and coordination capacity at the point of entry, capacity of provision of health services and transportation of sick travellers, training and knowledge sharing, and staffing and appropriate provision of health services logistics. Follow up probing questions were given by the interviewers during data collection sessions.

All interviews were conducted in the offices of the participants while ensuring a private and secure environment. The focus group was conducted in a meeting room in the health department at the airport. Interviews lasted for a minimum of 30 min, while the focus group lasted for an hour. Data saturation was not discussed with the participants. Data collection was ended after interviewing all stakeholders and reviewing available policy documents.

Data coding was performed by two of the authors (IG and KH). Inductive thematic analysis was conducted by reading the responses to develop codes. Furthermore, codes were compared to identify linked codes. Similar codes were classified within themes. Major themes were further divided into subthemes. Microsoft Office Word 2007 Tables were used to facilitate coding the study findings. Selected quotations were used to illustrate respondents’ views on particular issues.

Research ethics approval was obtained from the Research Ethics Committee at the College of Medicine, King Saud University, Saudi Arabia. No consent forms were signed by the participants. However, oral consent was provided, and approval to conduct the study was granted by the Directory of Public Health in Almedinah Almonawarah, and from the airport administration. Given the small sample size of this study and to reduce the risk of identifying the participants of this study, anonymity of the participants was secured when stating relevant quotations.

## Results

Data collection was conducted via several approaches. One focus group with the airport’s key public health professionals was conducted. Seven interviews with key informants related to airport administration, airport operating company, ground services company, immigration authority, security authority and customs authority were performed. Thirty-one policy documents and communications concerning public health measures applied at the airport and produced during the last 4 years were reviewed. All of the interviewed subjects were males, as female workers were a minority at the airport and all of the airport’s stakeholders were males.

**Fig. 1:**
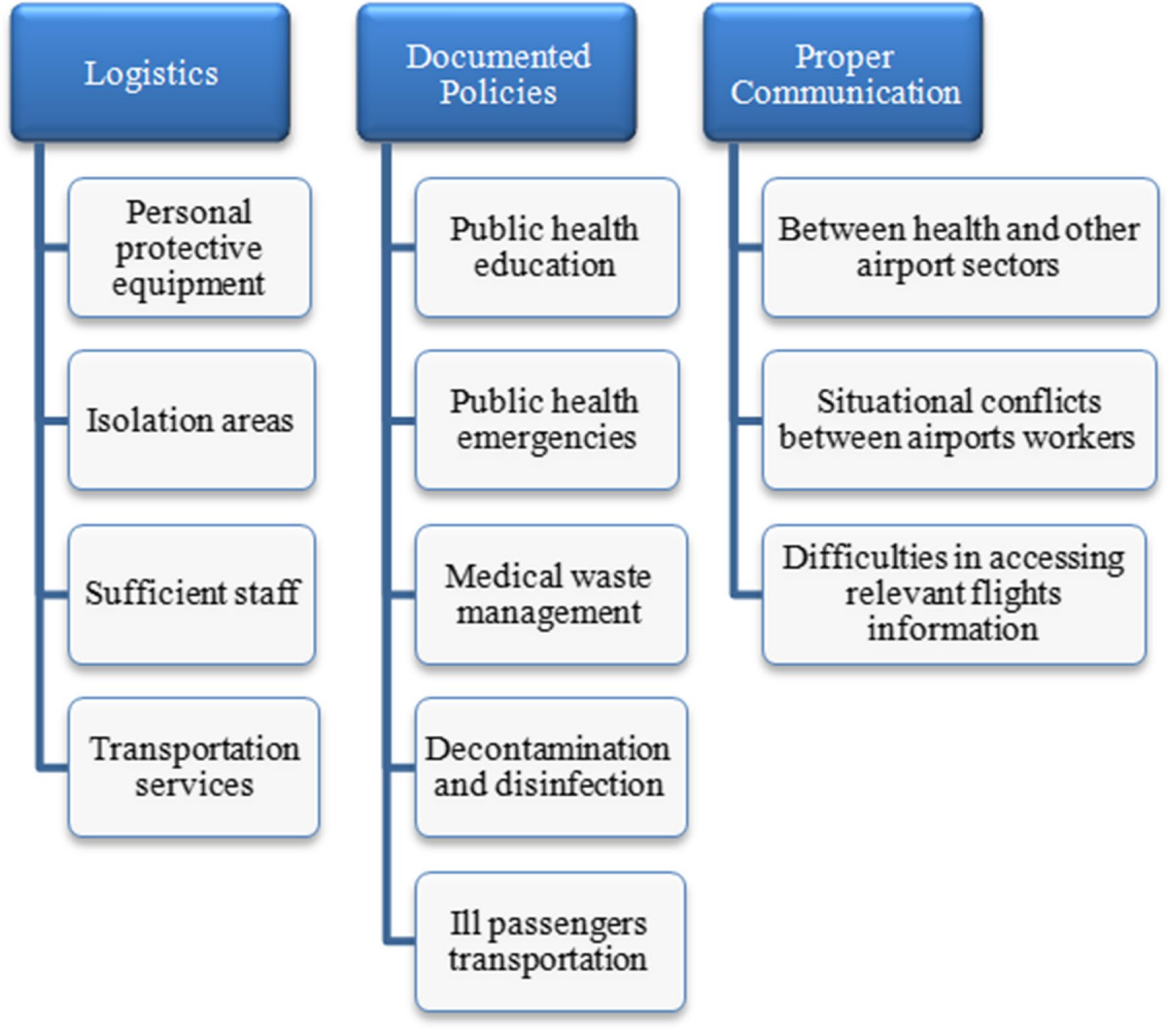
Main themes and subthemes summarizing issues concerning application of public health emergency measures at the airport

Data analysis produced 49 codes, three main themes and 12 subthemes. A summary of reported codes and their frequencies are presented in Table 1. The main themes and subthemes of study are summarised in Fig. 1. A description of main themes of the study is stated below.

**Table 1:** Reported major difficulties affecting public health emergencies interventions

Difficulties	Frequency of the code
Lack of a documented public health education policy for the airport workers	7
Difficulty in communication between the health sector and other airport sectors	7
Lack of documented protocol, guidance, or policy for public health emergencies	7
Shortage of Personal Protective Equipment	6
Difficulty in accessing or lack of documented protocol, guidance, or policy for dealing with spilled biological materials and medical waste management	3
Situational conflicts between health workers and workers of other sectors	3
Provision of materials and equipment needed for management of medical waste and disinfections	3
Lack of documented protocol, guidance or policy for decontamination and disinfection of passengers transporting vehicles and airports terminals	3
Lack of isolation area at the airport	2
Difficulties in movement of ambulances between aircraft and regional health facilities due to applied security measures at the airport	2
Shortage of appropriate size of the Personal Protective Equipment	1
Lack of protective glass at stations with frequent exposure to travellers	1
Refusal of passengers to take relevant vaccinations and prophylaxis	1
Miscommunication concerning updates of flight arrivals and flight schedules between health authority and the airport authority	1
Difficulties in application of screening measures against Ebola Viral Disease using screening cards due to shortage of health workers during certain shifts	1
Lack of documented protocol, guidance or policy concerning transportation of medical staff from the terminals to the aircraft	1

### Communication and coordination

Appropriate documentation was lacking. Similarly, appropriate methods of communication were lacking. The lacking of appropriate communication affected timely provision of preventive health services.

This was mostly apparent when one of the participants explained how a lack of communication between the airport’s control tower and the relevant health authority affected provision of relevant preventive services.

“According to the Ministry of Health regulations, we were instructed to provide vaccination and prophylaxis to the pilgrims arriving from selected countries. Once, a change in the flight schedule occurred without informing us and subsequently, we were not able to provide preventive services to all the flights’ passengers. Many pilgrims exited the airport without receiving required vaccinations or prophylaxis.”

Another indication of miscommunication between the health authority of the airport and the remaining sectors was due to a lack of involvement of the health authority representatives in the Command and Control Centre of the airport, as indicated in the following quote:

“The Central Command and Control at the airport does not report public health emergencies to the health authority at the airport (in a) timely manner since there is no representative of the health sector in the Command and Control Centre. Meanwhile, all other airport sectors have representatives in the Command and Control Centre.”

Several education materials and guidelines were found among the studied policy documents. For example, one of the documents has detailed information about the Ebola Virus Disease including prevention methods. That document was produced by the Saudi Ministry of Health and was supposed to be shared with all airport workers. However, appropriate educational materials were not then shared with other airport sectors, which forced one of the airport stakeholders to give us the following comment:

“I did not receive any educative materials pertaining to the Ebola Virus Disease and how to protect us from contracting the infection from arriving pilgrims. My employees are quite anxious”

It can be clearly seen that the miscommunication witnessed in the airport is shared between all airport sectors. Several airport sectors are apparently not delivering necessary information to the health authority. Similarly, the health authority is not appropriately responding to other sectors as indicated in the following quote:

“We tried contacting the Ministry of Health representatives at the airport concerning a health issue and we received no response during the last eight months.”

### Provision of documented policies

The lack of appropriate guidelines and protocols concerning public health issues at the airport was apparent in several areas. Unavailability of documented policies made application of preventive services confusing to the airport workers as indicated in these quotes:

“It is not quite clear what to do when there is a spill of blood or vomit in an airport terminal or in a transporting bus. Who should clean and disinfect the location, cleaning workers or health care workers?”

“Transporting sick travellers to (a) nearby hospital is not clear. Who should do it? (The)Airport ambulance or the National Ambulance Service?”

Similarly, specific guidelines have to be produced for specific clinical situations. In the following quote, it was apparent that the airport health authority does not have guidelines to make a decision about eligibility of sick travellers for air travel.

“A traveler with (a) health condition deteriorated suddenly in the airport. We needed a decision concerning the eligibility of the sick passenger for air travelling. However, the attending doctor was not able to make this decision.”

### Logistics

Several participants indicated shortage in the provision of logistic requirements needed for application of public health measures at the airport. This is an organisational deficiency stemming from a lack of orientation about the importance of public health at point of entry. The following quote illustrates how difficult it was to convince the airport authority about application of preventive measures:

“It was very difficult to convince the airport administration to establish screening points at the airport gates. However, the epidemic of Middle East Respiratory Syndrome in the country created awareness within the airport administration about the importance (of) public health emergency measures and it was allowed to install screening points.”

Additionally, health workers indicated how transportation services from the airport terminals were not properly provided. This lack of transportation services affected the provision of health services, which subsequently affected flight schedules, as apparent from the following quotes:

“On several occasions, we had to walk for long distances from the airport terminal to the aircraft. Having to walk for this distance affects our ability to deliver urgent health services in (a) timely manner.”

“We frequently face delays in flight schedule due to the late arrival of health care workers to aircrafts.”

The appropriate application of public health measures necessitates the need for sufficient staff and needed equipment. However, several participants indicated a lack of appropriate staffing, monitoring, and provision of Personal Protective Equipment, as one of the main obstacles, as stated in the following quotes:

“I remember once we needed an ambulance to transport a case from the airport. We were surprised to learn that the ambulance driver was not available.”

“We have no clear system of delivering Personal Protective Equipments. Sometimes we ask the airport’s health workers to give us masks and gloves on (a) personal basis.”

“We keep asking for vaccinations, prophylaxis and there is a delay in the delivery from the Ministry of Health.”

## Discussion

The findings of this study highlight the main factors affecting proper application of public health measures. These factors are related to a lack of properly documented communications and coordination between the airport sectors and regional health facilities, lack of protocols and guidelines including Public Health Emergency Contingency Plan (PHECP), and shortage in logistics and staff compared to the volume of travellers at the airport during the Hajj season. Reviewing policies and communication documents produced during the last 4 years and based on the findings of the interviews, it could be argued that these hindering factors are persistent and likely to continue in the subsequent seasons if not promptly addressed.

The situational analysis performed by this study was followed by the production of a PHECP. The production of this plan was a method of giving feedback to the stakeholders who were interviewed during data collection. The plan was designed to fill the gaps in knowledge and procedures concerning public health detected at the airport. Construction of the plan was produced in accordance with International Health Regulations guidelines and relevant international aviation authorities [1, 4–6]. Additionally, the plan aimed to ensure proper documentation of public health emergency procedures, documenting responsibilities of airport stakeholders and documenting channels of coordination between stakeholders.

For any PHECP to be effectively implemented, a Public Health Emergency Team (PHET) should be established at the airport to ensure the provision, testing and updating of the plan, and the sharing of relevant information with all stakeholders. Proper organization of health workers at the airport was proposed to enhance the efficiency of performed health services.

To facilitate implementing the plan, specific units within the team were proposed. These units should be specialized in rapid response actions for any public health events. The planning and intelligence unit is concerned with construction of specific urgent scenarios based on the epidemiological situation of the airport. Collaboration between finance administration and logistics provision services should be established to ensure efficient provision of vaccines, prophylaxis and suitable Personal Protective Equipment either during normal or urgent situations. A communication and coordination unit within the team should facilitate channels of communication between health workers at the airport, relevant stakeholders and relevant advisory units for specialized technical advice. A unit can be composed of several individuals or a single person.

**Fig. 2:**
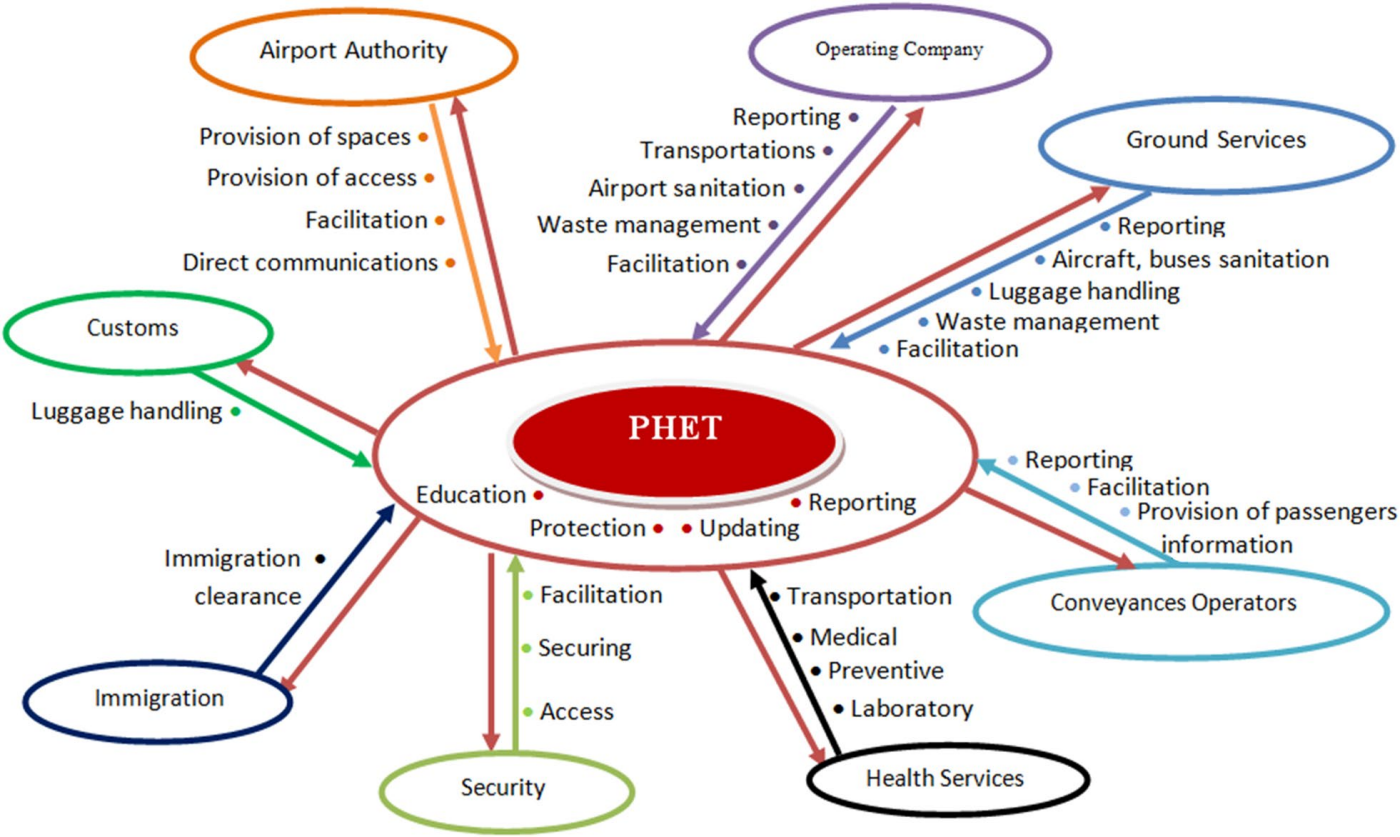
Description of shared responsibilities of airport sectors

Services provided at airports are the result of a complex network of activities performed by several governmental and private sectors. A summary of these activities is presented in Fig. 2. Handling any public health emergency in an effective and timely way cannot be accomplished without complete coordination and cooperation between all airport sectors. To enhance the cooperation between sectors, responsibilities and rights of each sector shall be agreed, documented and updated regularly.

Most of the witnessed conflicts that occurred between airport workers during the Hajj season of 2014 were mainly limited to minor verbal confrontations and loud arguments. The occurrence of these conflicts is likely to be caused by lack of communication and coordination between the stakeholders and the absence of specific documents to control areas of conflict. To resolve this issue, the production of documented Memoranda of Understanding (MoU) was proposed between all stakeholders. MoU were mainly proposed to enhance organizational collaboration between the stakeholders and airport workers. Around 6 MoUs were proposed where each one detailed the responsibilities and rights of participating partners. Additionally, proposing production of MoUs initiated a chain of communications between all airport stakeholders aiming to establish specific procedures conducted in particular situations such as, but not limited to, transportation of the ill and health workers, dealing with corpses, disinfection and decontamination.

It was proposed that the PHECP be performed not only in urgent situations, but also on a continuous basis that guarantees a comprehensive and rapid response when a public health emergency is announced at the airport. In general, the plan should include three phases, the preparedness phase, early warning phase and response phase.

The plan should ensure a competent preparedness status all year round and does not need to be triggered by any event. Additionally, subsequent preparatory and specific actions should take place to strengthen the readiness for a specific public health emergency being announced during the early warning phase. Finally, the plan should clearly indicate how to respond to very specific events occurring in any place in the airport that is related to the current epidemiological situation. The overall structure of the plan is summarized in Table 2.

**Table 2:** Summary of the PHECP

Preparedness phase	Early warning phase	Response phase
At all times	Global or national outbreak announcements	Imminent event report
Awareness raising Provision of facilities Provision of equipments Provision of vaccinations and medications Updating of PHECP Drill exercises Updating protocols Provision of MoUs and update Updating of communication lists Training Routine sanitation	Information gathering and sharing Scenarios construction Urgent provision of PPEs, vaccine, and prophylaxis Screening Education and training	Emergency announcement Urgent communications Rapid response team Preventive actions taken by stakeholders Response facilitation by relevant stakeholders Reporting and surveillance

According to our knowledge, this is the first qualitative case study conducted at an airport in Saudi Arabia to evaluate applied public health emergency systems. During the year 2014, the Saudi Arabian Ministry of Health was prepared to encounter the possibilities of a population spread of MERS-CoV and Ebola Viral Disease. Several plans were produced by the Ministry of Health with specific guidelines and procedures implemented on either clinical or non-clinical settings [7–9]. However, no organizational effort was implemented before this study to evaluate public health emergency systems at points of entry.

The main strength of this study is mainly based on utilizing a qualitative approach as an investigation tool. Utilizing a quantitative approach would not have allowed the collection of information rich in content and the validity of the data would have been questionable. Additionally, targeting stakeholders and decision makers allowed examining the main operational gaps and allowed benefiting the airport community with feedback detailed in the PHECP. In general, health investigations applied at airport settings in Saudi Arabia are limited and are mainly quantitative, such as the study by Al-Ghamdi and Kabbash [10].

The main methodological limitations of this study were mainly based on the inability to perform audio recording of the interviews and note taking was used as a recording method. This was mainly due to the sensitivity of the issues discussed and the fact that the key informants were decision makers at the airport. Another limitation was mainly related to the inability to recruit key informants related to the conveyances operators, as the stakeholders were not available at the airport.

## Conclusion

Several operational defects were detected at Prince Mohammed International Airport in Almedinah concerning applied public health emergency system. Situational analysis was performed during this study and a PHECP was proposed as a method of handling detected operational defects.

## Abbreviations

MERS-CoV: Middle East Respiratory Syndrome-Coronavirus
MoUs: Memoranda of Understanding
PHECP: Public Health Emergency Contingency Plan
PHET: Public Health Emergency Team

## References

[1] WHO. International Health Regulations 2005; 2008 (cited 2014 10th of September). http://www.who.int/ihr/9789241596664/en/.

[2] Saudi MoH. Health Regulations for Travelers to Saudi Arabia for Umrah& Pilgrimage (Hajj)-1435 (2014) 2014 (cited 2014 10th of September). http://www.moh.gov.sa/en/Hajj/HealthGuidelines/HealthGuidelinesDuringHajj/Pages/HealthRegulations1435.aspx.

[3] WHO. Assessment tool for core capacity requirements at designated airports, ports and ground crossings 2009 (cited 2014 10th of September). http://whqlibdoc.who.int/hq/2009/WHO_HSE_IHR_LYO_2009.9_eng.pdf?ua=1.

[4] WHO. Guide for Public Health Emergency Contingency Planning at designated points of entry 2012 (cited 2014 15th September). http://www.who.int/ihr/publications/9789290615668/en/.

[5] IATA. Public Health Emergency 2009 (cited 2014 10th of September). http://www.iata.org/whatwedo/safety/health/Documents/airlines-erp-checklist.pdf.

[6] Board TR. Infectious Disease Mitigation in Airports and on Aircraft 2013 (cited 2014 15th of September). http://www.trb.org/Main/Blurbs/169466.aspx.

[7] Saudi MoH. Infectious Diseases (MERS-CoV and Ebola) Diversion Plan for Hajj 2014 (cited 2014 19th November). http://www.moh.gov.sa/en/Hajj/HealthGuidelines/HealthGuidelinesDuringHajj/Pages/HealthRegulations1435.aspx.

[8] Saudi MoH. Infection prevention and control guidelines for patients with Middle East Respiratory Syndrome Coronavirus (MERS-CoV) infection 2014 (cited 2014 10th of September). http://www.moh.gov.sa/en/CCC/Regulations/Final-MERS-CoV%20IPC%20Guidelines-24.06.2014.pdf. 25129197

[9] Saudi MoH. INFECTION CONTROL GUIDELINES FOR MANAGEMENT OF SUSPECTED OR CONFIRMED EBOLA VIRUS DISEASE (EVD) 2014 (cited 2014 10th of September). http://www.moh.gov.sa/_layouts/FinalEVDIPCGuidelines08.08.2014.pdf.

[10] Al-Ghamdi AS, Kabbash IA (2011). Awareness of healthcare workers regarding preventive measures of communicable diseases among Hajj pilgrims at the entry point in Western Saudi Arabia. Saudi Med J.

